# Childhood TB sequel: evaluating respiratory function after treatment for pulmonary tuberculosis in a prospective cohort of Gambian children – a study protocol

**DOI:** 10.1186/s12890-023-02659-2

**Published:** 2023-10-12

**Authors:** Esin Nkereuwem, Schadrac Agbla, Muhammed Lamin Jatta, Uma Masterton, Olumuyiwa Owolabi, Victory Fabian Edem, Beate Kampmann, Toyin Togun

**Affiliations:** 1 Vaccines and Immunity Theme, MRC Unit The Gambia at the London School of Hygiene and Tropical Medicine, Atlantic Boulevard, Fajara, The Gambia; 2 Faculty of Infectious and Tropical Diseases, London School of Hygiene and Tropical Medicine, London, UK; 3 Department of Health Data Science, University of Liverpool, Liverpool, UK; 4 Department of Infectious Diseases Epidemiology, London School of Hygiene and Tropical Medicine, London, UK; 5 Charité Centre for Global Health, Institute of International Health, Charité Unversitatsmedizin, Berlin, Germany; 6 TB Centre, London School of Hygiene and Tropical Medicine, London, UK

**Keywords:** Childhood tuberculosis, Post-tuberculosis, Impairment, Sequelae, Lung disease, Spirometry

## Abstract

**Background:**

1.2 million children under 15 years are estimated to have developed tuberculosis (TB) in 2021. 85% of paediatric patients achieve successful treatment outcomes if treated for the first episode of TB. However, despite so-called successful treatment, TB leaves many survivors with permanently destroyed or damaged lungs. Data from prospective paediatric cohorts to establish the burden and evolution of post-TB lung disease (PTLD) are still absent. The *Childhood TB Sequel* study aims to describe respiratory consequences associated with pulmonary TB in Gambian children, describe the evolution of these sequelae, and determine associated epidemiological risk factors.

**Methods:**

We aim to recruit up to 80 subjects aged 19 years and below who have recently completed treatment for pulmonary TB. Recruitment started in April 2022 and is expected to continue until June 2024. Clinical assessment, chest X-ray, and comprehensive lung function assessment are carried out at treatment completion and again six and 12 months later.

**Discussion:**

The *Childhood TB Sequel* study will address existing research gaps to enhance our knowledge and understanding of the burden of PTLD in Gambian children. The study will also contribute to formulating a plan for post-TB evaluation and long-term follow-up strategies.

**Trial registration:**

ClinicalTrials.gov: NCT05325125, April 13, 2022.

## Background

In 2021, an estimated 10.6 million people fell ill with tuberculosis (TB), of which 1.2 million were children under 15 years [1]. Approximately 80% of these childhood TB cases affect the lungs [1]. However, from an optimistic standpoint, more than 85% of persons who receive treatment for the first episode of TB have a successful outcome, defined as smear or culture conversion to negative or clinical improvement on anti-tubercular treatment [1]. This success in treatment has resulted in about 155 million TB survivors as of 2020, of which 12% were children below 15 years of age [2].

The care for patients diagnosed with TB often ends at treatment completion without any further consultations to evaluate their health and well-being [3]. However, even after successful treatment, which is traditionally classified as either ‘cured’ or ‘treatment completed’, [4] pulmonary TB could permanently destroy or damage the lungs and progress from a treatable communicable disease into a chronic, non-communicable illness across the life course [5]. Moreso, there is increasing evidence that TB survivors continue to experience long-term sequelae that impact their physical and mental health, households, and communities even after successful treatment [5].

Although a growing literature describes a wide range of post-TB sequelae in adults, the actual burden and spectrum of post-TB lung disease (PTLD) remain poorly described in children. Our group previously documented that 52% of childhood TB survivors continued to experience recurrent respiratory symptoms beyond six months after treatment completion [6]. We also reported significantly reduced lung volumes and health-related quality of life in the childhood TB survivors compared to a healthy comparison group. Similarly, a prospective cohort study in Cape Town, which followed children from birth until five years of age, found that children who developed TB were more likely to wheeze consistently and had reduced anthropometry and lung function parameters in the post-TB period compared to children who never had TB [7].

The impact of TB on the lungs of children may be subclinical. Hence more detailed prospective paediatric data using lung function measurements regardless of symptoms and social determinants for lung health are urgently needed to establish the burden and evolution of PTLD in children [8]. The *Childhood TB Sequel* study seeks to add to the growing body of evidence by generating data describing respiratory symptoms, radiological abnormalities and lung function impairment in children after completing pulmonary TB treatment in The Gambia. The study will also assess how abnormalities evolve over the period of 12 months after treatment completion. This study includes adolescents aged 15 to 19 who have a considerably high incidence of TB and are often neglected in childhood TB research [9].

## Methods/design

### Study aims

The *Childhood TB Sequel* study aims to measure the proportion of Gambian children with long-term adverse outcomes associated with pulmonary TB, to describe the evolution of these sequelae, and to determine the epidemiological risk factors associated with these sequelae.

### Study setting

This study is set in the Greater Banjul Area (GBA) of The Gambia, which has mixed urban, peri-urban and rural populations [10]. In 2021, the incidence of TB in The Gambia was estimated to be 149 per 100,000 population [1]. Over 70% of all cases of TB notified in the country reside in the GBA [11]. Children aged 19 years and below comprise about 15% of all notified TB cases annually [1, 12].

After receiving a diagnosis of bacteriologically confirmed or clinically diagnosed (unconfirmed) pulmonary TB by public or private health providers, individuals are referred to their preferred Gambia National Leprosy and Tuberculosis Programme (NLTP) treatment clinic. The treatment clinics in different parts of the country are run by designated Leprosy and Tuberculosis Inspectors (LTI) who provide TB treatment, monitor treatment adherence, and assess the outcome at treatment completion. There are 20 TB treatment clinics in the GBA, which serve a population of about 700,000 [13].

### Study design and participant eligibility

The *Childhood TB Sequel* is an ongoing prospective cohort study in the GBA of The Gambia. Children aged 19 years and below who are in the final month of their treatment for pulmonary TB at any of the NLTP treatment clinics in the GBA are identified for possible inclusion in the study. After treatment completion and outcome classification by the LTI, each study participant is eligible if they meet all of the inclusion criteria and none of the exclusion criteria summarised in Table 1.

**Table 1:** Inclusion and exclusion criteria

Inclusion criteria	Exclusion criteria
Aged 19 years and below on the date of treatment completion	Relocate from the study area during the follow-up period
Residing within the Greater Banjul Area	Develop recurrent* pulmonary TB during the follow-up period
Previously diagnosed with either confirmed or unconfirmed pulmonary TB	
Have completed treatment with an outcome of *cured* or *treatment completed* within the preceding six weeks	
For children below 18 years, parent/caregiver willing to provide written informed consent as well as assent	

*either a true relapse or a new episode of TB caused by reinfection [4]

### Recruitment and procedures

Recruitment commenced in April 2022 and is expected to continue until June 2024. The study duration is 12 months, comprising an initial baseline (enrolment) visit and two follow-up time points at six months and 12 months from the enrolment date. A symptom screening will also be conducted via telephone at three and nine months. Table 2 shows the study time points and schedule of events (SOE). We aim to follow-up all children from baseline up to 12 months, regardless of their respiratory function.

We will evaluate clinical, radiological, spirometry and functional capacity measurements during the study visits according to the SOE in Table 2. Finally, we will characterise the changes in lung function over a period of 12 months after treatment completion.

**Table 2:** Schedule of Events (SOE)

	Baseline	Month 3^#^	Month 6	Month 9^#^	Month 12	Unscheduled visit
Current symptoms	X	X	X	X	X	X
Clinical assessment	X		X		X	X
Anthropometry	X		X		X	X
Physical examination	X		X		X	X
Chest x-ray	X		X		X	ICI
Spirometry	X		X		X	ICI
6-minute walk test	X		X		X	
Respiratory sample* for Xpert MTB/RIF Ultra	ICI		ICI		ICI	ICI

* Sputum (spontaneous or induced)/nasogastric aspirate

^#^ Conducted via telephone call

ICI: if clinically indicated

#### Clinical assessment

During the enrolment visit, we will obtain relevant clinical information, which will include current self- or parent-reported symptoms derived from the St George’s Respiratory Questionnaire, [14] a detailed history of the recent episode of TB for which they have just completed treatment, previous TB history, history of other known illnesses, smoking history and environmental exposure to smoking and biomass fuel. Additionally, we will conduct a general physical examination, nutritional assessment using anthropometric measurements, and detailed respiratory examination at enrolment, at six months and 12 months.

#### Radiological assessment

Per the SOE, we will obtain a chest X-ray (CXR) for each subject at enrolment, six months, and 12 months. Where available, radiographs obtained at TB diagnosis will also be reviewed and assessed for the classification of severity of TB disease as per the radiological case definitions [15]. The radiographs will be interpreted by two independent clinicians experienced in paediatric TB. A third reader will be used to resolve discrepancies in cases where there is a disagreement.

The radiological features of paediatric pulmonary TB are different from adults. In children, the disease is less likely to cause lung cavities commonly seen in adults and adolescents [16]. We expect the severity and typical patterns of post-TB radiological features to vary widely and to be related to the age and the radiological extent of the disease at diagnosis.

#### Lung function measurement

Lung function assessment will be performed for all children above four years by a trained technician using an Easy on-PC portable spirometer (ndd Medical Technologies, Zurich, Switzerland). Spirometry will be performed according to the American Thoracic Society (ATS)/European Respiratory Society (ERS) guidelines [17]. Briefly, we will record the ambient temperature, humidity and altitude prior to daily data collection. Subsequently, the spirometer will be calibrated using a 3 L syringe to ensure that measured volumes are within 3% of the syringe volume. After assessing for contraindications, we will explain and demonstrate the procedure. We will then ask the participant to perform up to eight forced exhalations while sitting. The procedure is repeated for all participants after receiving a bronchodilator (BD), salbutamol, via a spacer device.

Two reviewers will independently assess each spirogram for acceptability and repeatability and agree on a consensus result where there is discordance. Only spirometry traces that meet the ATS quality criteria will be included in the analysis. We will then record the z-scores for the highest forced vital capacity (FVC) and forced expiratory volume in 1 s (FEV1) and use this for the analysis. The Global Lung Function Initiative (GLI) 2012 reference equations will be used as the reference standard for spirometry data analysis, where FEV1, FVC or FEV1/FVC ratio below the lower limit of normal (LLN) will be deemed abnormal for the participants (Table 3) [18].

#### Functional capacity measurement

The six-minute walk test (6MWT) is a standardised measure of functional capacity or changes in functional capacity due to intervention in patients with lung disease [19]. The 6MWT will be assessed for all children above four years at the baseline, month 6, and month 12 study visits. The 6MWT will be performed according to the ATS guidelines [19]. In brief, the test will be conducted along a 30-meter-long, flat corridor with marked distances and turnaround points. Before starting the test, the participant will be requested to sit and rest for at least 10 min. After recording the baseline vital signs, dyspnoea and fatigue level will be documented using the Borg scale [20]. Afterwards, we will ask the participant to walk back and forth around the turnaround points. The objective is for the participant to walk as far as possible for six minutes, exerting themselves and taking rests as needed. At the end of six minutes, we will record the distance (in meters) covered by the participant, vital signs and fatigue level.

### Outcomes and case definition

The primary outcome for this study is PTLD at enrolment, at six months and 12 months after enrolment. Post-TB lung disease, as proposed during the first international post-tuberculosis symposium, will be operationally defined as *“evidence of chronic respiratory impairment in an individual previously adequately treated for pulmonary tuberculosis in whom active tuberculosis is excluded and in whom no other cause of chronic lung disease is the predominant cause”.* [5].

Evidence of chronic respiratory impairment will be assessed using self- or parent-reported chronic or recurrent respiratory symptoms derived from the St George’s Respiratory Questionnaire, [14] lung function measured by spirometry, and CXR occurring alone or in combination (Table 3). The evolution and pattern of change over the 6-monthly intervals will be assessed and classified as ‘improvement’, ‘no change’ or ‘deterioration’ based on pre-defined minimum clinically important difference (MCID) cut-offs for the measured parameters.

**Table 3:** Post-TB lung disease measurement parameters

Parameter	Abnormality signifying PTLD
Self- or parent-reported chronic or recurrent symptoms	Presence of any symptom
Lung function	*z*-score below the lower limit of normal (-1.64)
• Forced vital capacity (FVC) • Forced expiratory volume in 1 s (FEV1) • FEV1/FVC	
Chest X-ray	Presence of any TB-related abnormalities

*PTLD: post-TB lung disease

### Sample size

Over the past five years, the annual average notification of childhood and adolescent pulmonary TB in the GBA of The Gambia is 90. Assuming a population prevalence of the primary outcome, abnormal lung function, of 38.5%,^6^ and a finite population size of 90, a sample size of 73 cases allows the estimation of the proportion of children who develop post-TB lung function impairment in one year with 95% confidence and a margin of error of less than 5%. To allow for attrition, we will aim to enrol at least 80 children and adolescents. We expect at least 28% of all enrolled children to have had microbiologically confirmed TB [21]. We aim to follow-up all children from baseline up to 12 months, regardless of their respiratory function.

### Statistical analysis

The burden of children with the primary outcome, abnormal lung function, at baseline, at six months and 12 months after pulmonary TB treatment completion will be estimated (along with the corresponding 95% confidence interval) overall and stratified by confirmed versus unconfirmed pulmonary TB sub-groups. A pairwise comparison between the baseline and 6-month data and between the baseline and 12-month data will be made using McNemar’s test for categorical variables and the sign rank test for continuous variables. Linear mixed effects and logistic models will be used to estimate predictors of abnormal lung function over time. The impact and form of influence of the variables will be explored graphically and by calculating proportions or quantiles depending on the nature of the data.

### Ethical considerations

To ensure adherence to internationally accepted ethical standards, including the Declaration of Helsinki and the WHO Handbook for Good Clinical Research Practice, we have taken several measures in conducting our study [22]. First, the study protocol, informed consent document, and other study documents have undergone approval processes by The Gambia Government/MRC Joint Ethics Committee (Ethics Ref: 22,613) and the Observational/Interventions Research Ethics Committee of the London School of Hygiene and Tropical Medicine (Ethics Ref: 22,613–2). Additionally, study participants’ parents/legal guardians must provide written informed consent, while children aged 12 years and above must provide written informed assent. To maintain the confidentiality of each participant, they are assigned a unique anonymous study identifier upon enrolment. Furthermore, we uphold transparency and accountability by submitting an annual progress report to the Ethics committee and funders.

## Discussion

The lack of provision in the current Gambian TB treatment guidelines for specific post-TB monitoring or treatment of TB-related sequelae in children or adolescents, as well as the absence of estimates regarding the burden of different spectrums of PTLD, highlights the need for research on long-term sequelae and associated risk factors. The *Childhood TB Sequel* study is designed to address these gaps and contribute to the growing body of evidence in this field.

In this study, we will measure the proportion of children with PTLD at different time points: at TB treatment completion, six months, and 12 months after TB treatment. We will also document the different presentations and phenotypes of PTLD in Gambian children and adolescents. Additionally, we will describe the evolution of and track the changes in respiratory symptoms, radiological abnormalities, and lung function impairment in these children following pulmonary TB treatment completion in The Gambia.

By following the participants for at least 12 months, we aim to report lung outcomes that reflect chronicity rather than impairments associated with current TB disease. The data generated from this study will contribute to consolidating the definition of paediatric PTLD and proposing timelines for post-TB evaluation. This information will be valuable for making informed decisions and recommendations for long-term follow-up.

It is important to acknowledge certain limitations in our study. The lack of objective clinical data on the presence and extent of pre-existing comorbidities may limit our ability to attribute the sequelae exclusively to previous TB. Additionally, we have no influence over participants’ adherence to anti-TB treatment, which may influence post-TB recovery [23].

In conclusion, the *Childhood TB Sequel* study aims to provide valuable data to enhance our understanding of the long-term impact of pulmonary TB on the health and well-being of Gambian children and adolescents. Through insights into respiratory symptoms, radiological abnormalities, and lung function impairment, this study will contribute to the post-TB evaluation and the development of appropriate long-term follow-up strategies.

## Data Availability

Not applicable.

## Abbreviations

TB: Tuberculosis
PTLD: Post-tuberculosis lung disease
GBA: Greater Banjul Area
NLTP: National Leprosy and Tuberculosis Control Programme
SOE: Schedule of events
CXR: Chest X-ray
ATS: American Thoracic Society
ERS: European Respiratory Society
FVC: Forced Vital Capacity
FEV1: Forced Expiratory Volume in 1 s
GLI: Global Lung Function Initiative
LLN: Lower limit of normal
6MWT: Six-minute walk test
MCID: Minimum clinically important difference
MRC: Medical Research Council
